# Reframing Aging: A Generation's Work

**DOI:** 10.1093/geroni/igaa057.2559

**Published:** 2020-12-16

**Authors:** Patricia D'Antonio

**Affiliations:** The Gerontological Society of America, Washington, District of Columbia, United States

## Abstract

The Reframing Aging initiative, led by GSA on behalf of the Leaders of Aging Organizations, is a long-term, grant-funded social change endeavor designed to improve – or “reframe” the public's understanding of what aging means and the many contributions older people bring to society. Using evidence-based research, the initiative seeks to teach advocates how to tell an effective story about aging that will promote positive perceptions of aging and reduce ageism. This session will explore GSA's efforts to address ageism through Reframing Aging and the policy implications of this important initiative and will include lessons learned from experts who are utilizing reframing aging in their teaching and practice.

